# Editorial: Multidrug Resistance in Cancer: Pharmacological Strategies from Basic Research to Clinical Issues

**DOI:** 10.3389/fonc.2015.00105

**Published:** 2015-05-11

**Authors:** Chiara Riganti, Enrico Mini, Stefania Nobili

**Affiliations:** ^1^Department of Oncology, University of Turin, Turin, Italy; ^2^Department of Experimental and Clinical Medicine, University of Florence, Florence, Italy; ^3^Department of Health Sciences, University of Florence, Florence, Italy

**Keywords:** cancer, multidrug resistance, P-glycoprotein, reversing strategies

Tumor drug resistance is the leading cause of chemotherapy treatment failure. One of the more relevant mechanisms is represented by multidrug resistance (MDR) that leads to a reduced cellular accumulation of drugs due to increased efflux out of cells by the overexpression of several ATP-dependent efflux pumps or transporters. These proteins belong to the ATP-binding cassette (ABC) family and the most studied of them is P-glycoprotein (P-gp). Interestingly, P-gp acts as an efflux pump for various structurally unrelated anticancer agents (1, 2).

The aim of this issue, that includes nine contributes, is to highlight mechanistic aspects of the P-gp functions, provide information on the development of *in vitro* MDR tumor models, and describe potential strategies to overcome MDR.

Sharom (3) highlights the involvement of P-gp in a complex relationship with its lipid environment, which modulates the behavior of its substrates and many functions of the protein (e.g., ATP hydrolysis, drug binding, drug transport). Recently, some important principles governing P-gp–lipid and substrate–lipid interactions, and how these affect drug binding and transport, have been shown. In some cells, P-gp is associated with cholesterol-rich microdomains, which may modulate its functions. It is well known that the protein has also been proposed to operate as a drug translocase or flippase, moving its substrates from the inner to the outer leaflet of the membrane. The ability of substrates and modulators to interact with P-gp may depend on their ability to flip–flop between membrane leaflets. Membrane fluidizers and surfactants may reverse drug resistance, likely via an indirect mechanism.

On the other hand, Fu (4) introduces an interesting issue describing the localization of P-gp not only on the plasma membrane but also in many intracellular compartments (i.e., endoplasmic reticulum, Golgi, endosomes, lysosomes). P-gp can rapidly traffic and recycle among the intracellular compartments and between cellular organelles and plasma membrane, mainly via the indirect endosomal pathway. A role of cellular factors, such as Rab GTPases, in P-gp trafficking and recycling is also suggested.

McDermott et al. (5) provide a very detailed guide to the decision-making process for the development and ongoing maintenance of drug-resistant cancer cell lines. Relevant issues, such as the choice of the parental cell line, the strategy of cell exposure to anticancer drugs that has to mimic chemotherapy that patients receive in the clinical practice, as well as dose optimization, are discussed. Interestingly, McDermott et al. explore the heterogeneity of drug-resistant cell lines in relation to P-gp highlighting the complexity in developing P-gp resistant tumor models.

Until now, the scientific history of MDR-reversing strategies has been characterized by a repeated series of failures: indeed, the pharmacological inhibitors of the ABC transporters, such as P-gp, MDR-related proteins (MRPs), breast cancer resistance proteins (BCRP), have not reached the sufficient specificity and efficacy to be translated into the clinical practice.

Recently, however, the design of ABC transporters inhibitors has been progressively more refined, e.g., the creation of small libraries of compounds starting from versatile scaffolds or the use of dual effect drugs or multitarget drugs (i.e., chemotherapeutic drugs that are chemically conjugated with an ABC transporters inhibitor) can increase the selectivity and potency of transporter inhibitors and modulators, as described in the topic of Zinzi et al. (6). Although very innovative, these approaches do not solve the crucial challenge in the field of MDR-reversing strategy, i.e., achieving the maximal efficacy and selectivity against MDR cells. Recent high-throughput screenings of pharmacological libraries identified specific compounds, such as compounds increasing the generation of reactive oxygen species (ROS) and depleting cells of the anti-oxidant metabolite glutathione (GSH), which were unexpectedly more effective in ABC transporter-overexpressing cells than in ABC transporter-negative cells. This phenomenon is known as collateral sensitivity (7). The oxidative-mediated collateral sensitivity is, however, a multifaceted event in resistant tumors. In this topic, Gauthier et al. (8) demonstrate, for instance, that the GSH depletion induces apoptosis in chemoresistant cells overexpressing MRP1, but not in resistant cells overexpressing BCRP. This means that although MDR cells are generally more damaged than chemosensitive cells by oxidative stress, the degree of this damage is highly dependent on the spectrum of ABC transporters expressed by each tumor. Increasing the GSH efflux via ABC transporters is not the only strategy that can increase ROS in MDR cells. Changing the activity of redox sensitive factors that control anti-oxidant enzymes, phase 2 detoxifying enzymes, and stress response proteins, such as nuclear factor (erythroid-derived 2)-like 2 (Nrf2), apurinic-apyrimidinic endonuclease 1/redox factor 1 (APE-1/Ref-1), and forkhead box O (FoxO), can achieve the same goal. Interestingly, these transcription factors not only control the redox balance but also regulate the expression of specific ABC transporters as reported by Polimeni and Gazzano (9). Therein, their targeting may result in pleiotropic chemosensitizing benefits against MDR tumors.

A second approach to improve the selectivity of chemosensitizing agents against MDR cells is the use of nanoparticles, for the active targeting of chemotherapeutic drugs, chemosensitizing agents or siRNA within the resistant tumor as proposed in this topic by Conde et al. (10). Several open questions need to be solved before the translation of nanoparticle-based approaches into the clinical practice, such as the biocompatibility and the long-term safety of nanoparticles. Once the first phase I–phase II clinical trials and observational studies have been concluded, these issues will be clarified and nanoparticles may become useful tools also for the delivery of collateral sensitivity inducers or agents targeting redox sensitive factors within MDR cells.

Cancer stem cells (CSCs) exhibit several mechanisms of resistance against anticancer drugs that are mainly represented by the expression of ABC transporters and activation of different signaling pathways (e.g., Wnt/β-catenin signaling, Hedgehog, Notch, Akt/PKB). Thus, compounds able to modulate MDR on CSC membranes could induce cytotoxicity in these cells, as described by Zinzi et al. (11).

Two models have been suggested to explain the connection between MDR and CSCs: the “original” MDR model according to which, after exposure to the chemotherapeutic agent, only CSCs expressing ABC transporters repopulate the tumor, and the “acquired” MDR model according to which after chemotherapy, only CSCs survive and this population of survival cells, after mutations, originates new and more aggressive drug-resistant cell phenotypes. Thus, the combination of CSC targeting agents with novel or conventional cytotoxic drugs could lead to a potentiated effect. An innovative multimodal strategy, i.e., an approach in which specific CSC targeting drugs exert simultaneously the ability to circumvent tumor drug resistance (by ABC transporter modulation) and to exert cytotoxic activity toward CSCs and the corresponding differentiated tumor cells, may be hypothesized.

Targeting ABC transporters is a challenge not only limited to tumors cells but it also involves the tumor microenvironment in specific districts: as demonstrated in this topic by Adkins et al., breast cancer cells metastasizing within central nervous system are rich of P-gp and are surrounded by a complex vasculature expressing P-gp as well (12). This situation, which is common to other metastatic cancers, creates “MDR niches,” making harder the full eradication of resistant cells and easier the tumor relapse. Finding compounds overcoming P-gp activity in both the tumor cell and the tumor microenvironment-associated cells is a future open challenge in the field of MDR-reversing strategies.

This topic has reviewed pharmacological strategies to overcome MDR. Fighting MDR involves multiple skills and know-how, including the ability to develop suitable *in vitro* drug-resistant tumor models, the understanding of the ABC transporter functions, the use of medicinal chemistry, the production of new nanomaterials, the analysis of the biochemical features of MDR cells, and the management of clinical trials. Such multidisciplinary approach is mandatory to open new perspectives against chemoresistant tumors.

## Conflict of Interest Statement

The authors declare that the research was conducted in the absence of any commercial or financial relationships that could be construed as a potential conflict of interest.
